# Two preclinical tests to evaluate anticancer activity and to help validate drug candidates for clinical trials

**DOI:** 10.18632/oncoscience.132

**Published:** 2015-02-20

**Authors:** Miguel López-Lázaro

**Affiliations:** ^1^ Department of Pharmacology, Faculty of Pharmacy, University of Seville, Spain

**Keywords:** Anticancer drugs, Cancer, Drug discovery

## Abstract

Current approaches to assessing preclinical anticancer activity do not reliably predict drug efficacy in cancer patients. Most of the compounds that show remarkable anticancer effects in preclinical models actually fail when tested in clinical trials. We blame these failures on the complexity of the disease and on the limitations of the preclinical tools we require for our research. This manuscript argues that this lack of clinical response may also be caused by poor *in vitro* and *in vivo* preclinical designs, in which cancer patients' needs are not fully considered. Then, it proposes two patient-oriented tests to assess in *vitro* and *in vivo* anticancer activity and to help validate drug candidates for clinical evaluation.

## INTRODUCTION

Many cancer patients are diagnosed with or develop distant metastasis. Despite the recent approval of numerous anticancer drugs [1], most of these patients do not overcome the disease. For example, over 50% of people diagnosed with lung cancer have distant metastasis at the time of diagnosis, and only 4% of them survive more than five years. The prognosis for patients with other common metastatic cancers is not much better. The five-year relative survival rate for patients with metastasis to distant sites is 28% in prostate cancer, 24% in breast cancer, 13% in colorectal cancer, 3% in liver cancer and 2% in pancreatic cancer [2]. Many patients diagnosed with these common cancers eventually die from the disease despite surviving five years after diagnosis. Because advanced cancers need to be treated with drugs, there is an urgent need to develop better pharmacological therapies.

The first step to developing better treatments is the selection of good compounds or strategies for testing. For this purpose, understanding the disease is important to find better ways to exploit its weaknesses. Given the limited progress made to date in the treatment of advanced cancers, questioning the established views and considering unconventional approaches may also be important. The aim of this research perspective is not to review or propose potential anticancer strategies.

Testing the drug candidates using robust preclinical models is the next crucial step to developing better treatments. These models should not only identify the best drug candidates, but should also predict whether their anticancer potential is high enough to deserve clinical evaluation. An inadequate preclinical design may result in the selection of poor candidates, which may lead to a substantial allocation of preclinical and clinical resources to ineffective compounds. An inadequate design may also result in a failure to identify new drugs that could change the lives of cancer patients.

Current preclinical models do not reliably identify the best drug candidate in a group of compounds [3]. For instance, the selected candidate from a library of over three hundred thousand compounds [4] was found to be more toxic to normal cells than to cancer cells [5,6]. These models do not reliably predict drug efficacy in patients either; most of the candidates that show efficacy in preclinical models actually fail when tested in clinical trials [7-9].

Researchers blame these failures on the complexity of the disease and on the limitations of the preclinical tools we require for our research [9]. Here I argue that this lack of clinical response may also be caused by poor *in vitro* and *in vivo* preclinical designs, in which cancer patients' needs are not fully considered. Then, I propose two patient-oriented tests to assess preclinical anticancer activity and predict drug efficacy in cancer patients more reliably.

### Anticancer potency in malignant cells and antitumor activity in animal models are poor predictors of clinical response

Many drug candidates are proposed for clinical evaluation based on their ability to target cancer cells at low concentrations and to induce antitumor activity in animal models. Despite its widespread use, this approach does not fully consider cancer patients' needs and may therefore result in a poor selection of drug candidates for clinical development.

Cancer patients need drugs that improve the efficacy of the current pharmacological treatments. Accordingly, drugs are not approved for cancer therapy unless they match or improve the efficacy (survival rate) of the standard treatments when tested in Phase III clinical studies. Testing whether or not the experimental treatment improves the survival rate of the standard treatment would be a reasonable way of assessing anticancer activity in animal models. Instead of assessing improvements in survival, researchers frequently evaluate the magnitude of the antitumor response induced by the drug candidate in rodent models. Since researchers often measure different parameters in animal and human studies, and since the magnitude of the drug response in rodents and humans may be rather different, it is not surprising that the responses observed in preclinical *in vivo* models are not reproduced in clinical trials.

Researchers assessing anticancer activity *in vitro* often misunderstand cancer patients' needs; this may result in a poor selection of drug candidates in the screening step of the drug discovery process [3]. Cancer patients do not need drugs that kill cancer cells at low concentrations. They do not need drugs that act on particular targets involved in cancer cell proliferation and survival either. They need drugs that kill their cancer cells at concentrations that do not significantly affect their healthy cells. If a drug targets cancer cells at low concentrations but also targets normal cells at similar concentrations, the highest drug doses tolerated by the patients will be insufficient to reach the tissue drug concentrations required to eliminate their cancer cells. If a drug kills cancer cells without significantly affecting healthy cells, it does not matter much its pharmacological targets or the concentration at which it kills the cancer cells. A reasonable way of assessing anticancer activity *in vitro* would be to test if the drug candidate improves the ability of the standard drugs to kill cancer cells without significantly affecting nonmalignant cells [3].

Instead of looking for compounds that improve the selectivity (*in vitro*) and the survival rate (*in vivo*) of the existing pharmacological treatments, researchers typically look for compounds that target malignant cells at low concentrations and induce antitumor activity in animal models. For example, Wang *et al*. [4] screened a library of 359,484 compounds and found that the cardiac glycoside bufalin was a potent inhibitor of the steroid receptor coactivators SRC-3 and SRC-1. Because bufalin could also inhibit the proliferation of human breast cancer cells at very low concentrations and induce antitumor activity in mice transplanted with human breast cancer cells, the authors discussed that this compound could have potential for cancer therapy [4]. Likewise, Denicolai *et al*. [10] screened a chemical library of 1,120 compounds and selected the cardiac glycoside proscillaridin A based on its ability to target glioblastoma cell lines potently (nanomolar concentrations). Proscillaridin A also induced anticancer activity in mice xenotransplanted with human cancer cells, and the authors suggested that this compound was a promising chemotherapeutic agent [10].

It is important to recognize that the ability of a compound to target cancer cells at low concentrations does not reliably predict its ability to kill cancer cells selectively. For example, the cardiac glycoside bufalin targets breast cancer cells at very low concentrations [4], but it is more cytotoxic to breast healthy cells than to breast cancer cells [5,6]. We have also observed that the most cytotoxic compound in a series of aziridine derivatives was 7 times more toxic to normal cells than to cancer cells, while a less potent compound of the series was over 50 times more cytotoxic to breast cancer cells than to several types of normal cells [11].

It is also important to note that the ability of a compound to induce antitumor activity in animal models does not reliably predict its ability to selectively kill human cancer cells *versus* human healthy cells. Rodent models can show the selectivity of a compound in human cancer cells *versus* rodent normal cells (xenograft models), or in rodent cancer cells *versus* rodent normal cells (allograft and spontaneous models). However, animal models cannot show if a compound can selectively kill human cancer cells *versus* human normal cells. This distinction is important to avoid experimental artifacts caused by species differences in drug sensitivity. For example, we have observed that rodent cells are extremely resistant (over 1000-fold) to the cytotoxicity of cardiac glycosides when compared to human cancer and nonmalignant cells [12]. It is therefore relatively easy to find a marked *in vivo* anticancer activity if one transplants human cancer cells into mice and then treats the animals with a cardiac glycoside such as bufalin or proscillaridin A [4,10,13-16]. This activity, however, is probably caused by the ability of these compounds to selectively kill human cells *versus* rodent cells rather than by their ability to selectively kill cancer cells [6,12,17-19]. The marked antitumor activity of these compounds in xenograft models is unlikely to be reproduced in clinical trials.

### Two patient-oriented tests to assess preclinical anticancer activity

**Figure 1:**
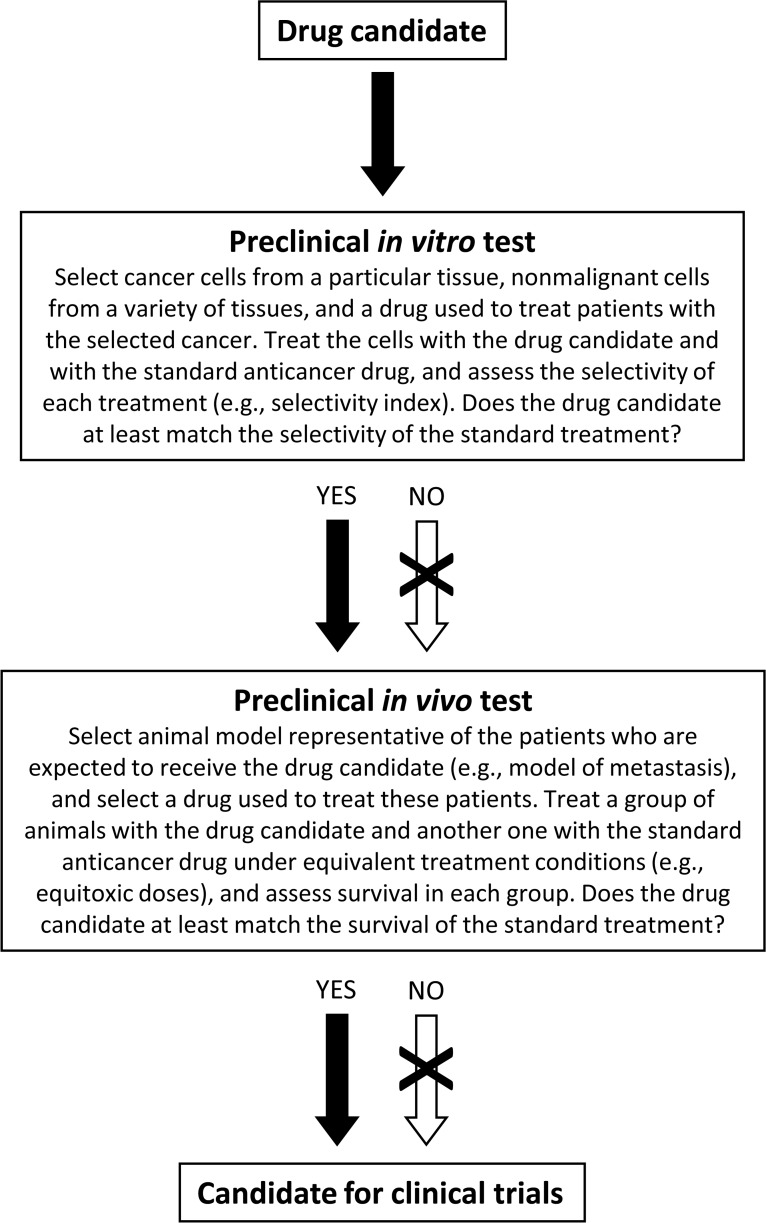
Basic protocol to assess preclinical anticancer activity It can be used as a standalone tool to evaluate the activity of novel compounds. The tests can also be used to help validate drug candidates that have shown efficacy in other preclinical tests. The experimental treatments and the standard treatments can be single drugs or drug combinations. The crossed-out arrows can be bypassed in specific situations (e.g., when the drug does not kill cancer cells through a direct cytotoxic mechanism or when the pharmacokinetics or pharmacodynamics of the drug in rodents and humans are not similar). See text for details.

These tests are designed to detect whether or not an experimental treatment is better than the standard treatment used in cancer patients. They can help researchers decide whether to propose a drug candidate for clinical evaluation. They can also be used as standalone tests to assess preclinical anticancer activity. They are based on setting suitable experimental conditions to answer two questions: Can the drug candidate improve the ability of the existing drugs to selectively kill cancer cells *versus* a variety of nonmalignant cells? Can the drug candidate improve the survival of the standard treatment when tested under equivalent experimental conditions in animal models representative of patients for whom the new drug is intended? A basic protocol that includes both tests is proposed in Figure 1.

The *in vitro* test has been described previously [3,20]. It consists of (1) exposing cancer cells and a variety of nonmalignant cells to the drug candidate and to the standard drugs used to treat patients with the selected cancer, (2) estimating cell viability with a simple cytotoxicity test (e.g., SRB assay, MTT assay), (3) calculating one or several cytotoxicity parameters (e.g., IC_50_, IC_90_, LC_50_) for each drug in each cell type, (4) calculating a selectivity index for each drug (e.g., dividing the IC_50_ value in the nonmalignant cells by that in the cancer cells) and (5) comparing the selectivity indices of the experimental drugs with those of the standard anticancer drugs [3,20].

Several aspects should be considered for implementing the *in vitro* test. The cancer cells and nonmalignant cells should be human to avoid unreal selectivity values caused by species differences in drug sensitivity. For example, if we test a cardiac glycoside in human cancer cells *versus* rodent normal cells, we will find a high selectivity caused by the resistance of rodent cells to the cytotoxic effects of these compounds [12]. Selecting nonmalignant cells from a variety of tissues is also essential for implementing this approach robustly. We should always keep in mind that a high toxicity to normal cells from just one particular tissue may cause that the maximum doses tolerated by the patients are insufficient to reach the drug concentrations required to eliminate their cancer cells. A selection of nonmalignant cells from tissues commonly affected by cancer pharmacotherapy would help us predict dose-limiting toxicity to normal tissues. Selecting nonmalignant cells from the same tissue than that of the cancer cells is also important to avoid unreal selectivity values originated by tissue differences in drug sensitivity. For example, if we are looking for drugs for brain cancer, we should select brain cancer cells and a variety of nonmalignant cells from several tissues including the brain. This would prevent us from choosing drug candidates that induce selective cytotoxicity towards a particular tissue (e.g., brain) but have a limited selectivity towards cancer cells (e.g., ellipticinium derivatives ) [21,22].

The *in vitro* test can be used to assess the anticancer potential of drug combinations. Currently, the therapeutic potential of a drug combination is typically evaluated by testing in cancer cells if its cytotoxicity is enhanced (potentiation factor) or synergistically increased (combination index) in relation to the cytotoxicity induced by each drug individually. This approach assumes that increasing the cytotoxic potency of a drug combination in cancer cells will also increase its therapeutic potential. The key feature of an efficient anticancer drug or drug combination is their ability to kill cancer cells selectively, and not their ability to kill cancer cells potently. A drug combination that induces a strong cytotoxic synergism in cancer cells will not be clinically effective if it induces a stronger synergism in nonmalignant cells. A drug combination that induces an antagonism in cancer cells may be clinically effective if it induces a stronger antagonism in nonmalignant cells. As discussed elsewhere, it does not matter whether a drug combination is synergistic, additive or even less than additive for cancer cells. What is important is that such a combination has a high selectivity towards cancer cells *versus* normal cells [23,24]. But there is something else to consider. The drug combination should not just be more selective than the standard drug or drug combination used to treat patients with the selected cancer. A drug combination that induces synergism in cancer cells and antagonism in nonmalignant cells will not be clinically useful if its selectivity towards cancer cells is much lower than that of the standard treatment. The *in vitro* test can reveal whether an experimental drug combination improves the selectivity of the standard drug or drug combination used in cancer therapy.

The preclinical *in vivo* test shown in Figure 1 seeks to establish the most suitable experimental conditions for detecting whether the drug candidate is better than the existing treatment. It seeks to mimic the test that every anticancer drug needs to pass to be approved for human use: phase III clinical trials. In these trials, the experimental treatments are compared with the current standard treatments. To pass this test and receive approval for use in cancer patients, the new drugs should be at least as effective as the existing anticancer drugs.

Improved survival is the standard endpoint to assess effectiveness in phase III trials. Because most cancers requiring pharmacotherapy are difficult to cure, prolonging patient survival is a major goal of cancer pharmacotherapy. In addition, the moment someone dies can be measured easily and unequivocally. Assessing survival does not depend on measuring the size of tumors that are sometimes difficult to measure or even locate (e.g., micrometastasis). Furthermore, survival accounts for any increase in the death rate due to long-term drug toxicity. Measurement of tumor shrinkage in response to treatment provides useful information; however, it is not a proof that a treatment is truly beneficial. Tumor shrinkage in response to treatment is the typical endpoint for phase II trials, but not for phase III trials. Because phase III trials are regarded as the most rigorous studies for testing whether new treatments work, improved survival should also be the standard endpoint to assess effectiveness in animal models.

In phase III clinical trials, the experimental treatment and the standard treatment are tested under the most favorable conditions. Although finding these conditions may be difficult, it is widely accepted that the drugs should be tested at the maximum tolerated dose (MTD) determined in phase I (or phase II) clinical studies. A phase III trial would never receive approval if the experimental drug were planned to be tested at the MTD and the standard treatment at one-half of the MTD. The match would not be fair. Selecting equitoxic drug doses should be equally important to assess drug efficacy in animal models. This is not always the case, however. The determination of MTD in rodents is somewhat different from in humans. Because mice cannot tell us if they are experiencing unacceptable side effects, higher doses are required to determine this parameter. A fraction of the MTD value (e.g., 0.375 x MTD and 0.25 x MTD: http://dtp.nci.nih.gov/branches/btb/acute_tox.html) could be used for implementing the *in vivo* test.

Patients with localized and resectable tumors are not eligible for phase III clinical trials evaluating the efficacy of new pharmacological treatments. The reason is that the participants of these trials should represent the population that would eventually receive the new drug, and these patients do not represent such a population. These patients are usually treated successfully with surgery and are not expected to benefit from the new treatment. Something that is unacceptable in human studies is however acceptable in animal studies. We commonly use mice with localized and resectable tumors to evaluate the efficacy of our experimental drugs. Although we could easily cure these mice by surgical resection of the tumors, we spend resources trying to find drugs that will never improve the efficacy of a treatment that we already have. Selecting animal models representing the patients who are expected to receive the new treatment seems to be a more reasonable approach to assessing anticancer activity *in vivo*. Because most patients requiring pharmacotherapy have metastatic disease, selecting animal models of metastasis should be a priority in most situations.

### Strengths and limitations of the tests

The most important strength of the tests is that they are designed to detect the type of drugs that cancer patients need. Cancer patients need drugs that improve the efficacy of the existing pharmacological treatments, and both tests measure whether or not the drug candidate improves the efficacy of these treatments. Establishing the best parameter to measure efficacy *in vitro* and *in vivo* is important. The tests propose that selectivity and survival are the best parameters to determine drug efficacy *in vitro* and *in vivo*, and establish experimental conditions to measure these parameters robustly.

Another important advantage of these tests is that they make our preclinical tools better. Depending on the cell lines or animal models we use for testing our drugs, the magnitude of the drug response may be rather different. This makes extrapolation and comparison of results problematic. It also makes it difficult to know how much effect we should observe to say that our drug is promising. Therefore, when the extent of the response in two models is not similar, we usually consider that these models may be inadequate for testing our drugs. For example, the NCI60 human tumor cell line anticancer drug screen did not include nonmalignant cell lines as controls because the normal cell types available at that time were either very resistant or very sensitive to the cytotoxic activity of the tested anticancer drugs [22]. The researchers involved in the development of the screen probably thought that the resistant normal cells would underestimate, and the sensitive normal cells would overestimate, the anticancer potential of the experimental compounds. The tests discussed in this manuscript make our preclinical tools better because they focus on whether the experimental drug improves the activity of the standard drug, and not on the magnitude of their effects in particular cell types or animal models. In these tests, it does not matter whether the selectivity of the drug candidate in particular cell types is 2, 10 or 100. What matters is if its selectivity is higher than that of the drugs used in cancer therapy. Those normal cell types that were considered to be inadequate for the NCI anticancer drug screen would be adequate for the *in vitro* test. With the *in vitro* and the *in vivo* tests, it is easy to know how much activity we should observe to say that our drug is promising: we should improve the activity of the standard anticancer drugs.

The implementation of these tests may lead to an important saving of research resources. For example, large amounts of resources have been dedicated to investigate the anticancer potential of the curry constituent curcumin. We have learned from thousands of research studies that curcumin modulates numerous molecular targets involved in cancer cell death and proliferation pathways. Curcumin can also kill many types of cancer cells *in vitro* and induce antitumor activity in xenograft models [25-27]. Several clinical trials have evaluated the anticancer activity of curcumin, and others are ongoing or recruiting participants (https://clinicaltrials.gov/). None of the completed clinical trials, however, has shown relevant anticancer effects so far. Unfortunately, none of the over 2,500 papers in PubMed that contain the terms “curcumin” and “cancer” has assessed under adequate experimental conditions whether curcumin improves the selectivity or survival of the standard anticancer drugs. A single research work assessing the selectivity of curcumin and of a variety of anticancer drugs in an adequate panel of cancer and nonmalignant cell lines could reveal that this huge amount of preclinical and clinical resources may not have been spent in the best interests of cancer patients. Since curcumin typically kills cancer cells in the 5-50 μM range [28], we evaluated its cytotoxic activity in lung cancer cells and lung nonmalignant cells at this concentration range and observed an identical cytototoxic profile in both cell lines [29]. The implementation of the protocol shown in Figure 1 would not only lead to an important saving of resources, but would also prevent many animals and patients from receiving ineffective drugs.

Advanced technologies are not required for implementing these tests. This is an important advantage for researchers with limited budgets. Any research team with cell culture and animal facilities can put these tests into practice and assess preclinical anticancer activity robustly.

These tests have limitations, however. The *in vitro* test has the typical limitations of all *in vitro* investigations. Because *in vitro* conditions do not represent *in vivo* conditions faithfully, drugs that work *in vitro* may not work *in vivo*, and vice versa. In addition, this test would not be suitable to assess the anticancer activity of drug candidates that do not kill cancer cells through a direct cytotoxic mechanism, such as immunostimulant compounds. Modified or alternative experimental conditions should be established for assessing the *in vitro* anticancer activity of these compounds.

Human cancers cannot be faithfully reproduced in mice. The following most important limitation of the *in vivo* test comes from the fact that the pharmacokinetics and pharmacodynamics of a drug in rodents may be different from in humans. In two species, the same drug may not only induce different effects or similar effects with different intensities, but may also be processed differently. These variations between species are commonly caused by differences in target cell sensitivity and in drug metabolism, and make extrapolations between rodents and humans difficult. As discussed previously, cardiac glycosides induce marked antitumor effects in mice transplanted with human cancer cells; this probably happens because human cells are much more sensitive than rodent cells to the cytotoxicity of these compounds. Experimental artifacts caused by species-differences in drug sensitivity could be prevented or detected by testing the cytotoxicity of the experimental drugs and the standard drugs in human cancer cells, human nonmalignant cells and rodent cells [12]. If the drug candidate improves the survival of the standard drug in rodent models, these cytotoxicity experiments would reveal if the survival improvement is caused by species-differences in sensitivity rather than by a selective anticancer effect. Species differences in drug metabolism may also lead to experimental artifacts. A standard anticancer drug may be metabolized in rodents much more extensively than in humans. Its activity in rodents would therefore be reduced, and it would be easier for an experimental drug to show a survival benefit. This benefit, however, would not occur in humans. The *in vivo* test could also underestimate the efficacy of drug candidates that are metabolized in rodents much more extensively than in humans. Measuring the levels of the experimental drugs and standard drugs in plasma and target tissues may assist researchers in detecting possible experimental artifacts caused by species-differences in drug distribution and metabolism.

## CONCLUDING REMARKS

Survival in patients with the most common metastatic cancers is low [2]. Because the main form of treatment for these patients is pharmacotherapy, many research resources are invested to develop new anticancer drugs. As a result, thousands of research papers assessing preclinical anticancer activity are published every year, but very few clinical successes are produced. Clinical trials in oncology actually have the highest failure rate compared with other therapeutic areas [7,9]. It is broadly accepted that the limitations of preclinical tools, such as inadequate cancer cell lines and mouse models, make it difficult for even the best scientists working in optimal conditions to make a discovery that will ultimately have an impact in the clinic [9].

This research perspective argues that the high failure rate of clinical trials in oncology may also be caused by an inadequate use of our preclinical tools. If researchers involved in cancer drug discovery (e.g., authors, reviewers and editors) do not use or request reliable preclinical tests for assessing anticancer activity, the problem of low clinical trial success rates will not be solved by providing the cancer research community with collections of well-characterized cancer cell lines, with novel animal models or with the most sophisticated technologies. Despite using these tools, researchers may still consider that a drug deserves clinical evaluation when it targets cancer cells at low concentrations and induces a marked inhibition of tumor growth in mice. If the cytotoxic mechanism of the drug is mediated by a novel therapeutic target and is unraveled with advanced technologies, this research work may well be published in a prestigious scientific journal, which may lead to a clinical trial with a high probability of failure.

Two simple and cost-effective tests for assessing preclinical anticancer activity are proposed in this manuscript. Because cancer patients need drugs that improve the efficacy of the existing treatments, the aim of both tests is to detect this type of drugs. After discussing that selectivity and survival are the best parameters to measure anticancer efficacy *in vitro* and *in vivo*, the tests establish experimental conditions for detecting these parameters robustly. Despite having limitations, these tests can help researchers assess the *in vitro* and *in vivo* anticancer activity of their compounds. They can also be useful for deciding whether or not a drug candidate deserves clinical evaluation. Clinical trial failure rates would probably decrease if the drug candidates had to pass both tests before reaching clinical trials. Drug candidates passing both tests should also show that their anticancer activity is not an experimental artifact caused by pharmacokinetic and pharmacodynamic differences between rodents and humans. A possible way of minimizing these artifacts has been proposed.

The following strategies would facilitate the implementation of these tests in the cancer research community. Cancer research organizations could take the lead in assembling and providing researchers with an adequate collection of cell lines (malignant and nonmalignant) and animal models for each type of cancer. They could also provide researchers with detailed *in vitro* and *in vivo* protocols to assess anticancer activity in these cancers. Results (e.g., selectivity indices and survival rates) obtained with the standard drugs under the experimental conditions of these protocols would help researchers know what we should match or improve. Researchers assessing preclinical anticancer activity should avoid experimental approaches that are poor predictors of activity in cancer patients (e.g., anticancer potency in malignant cells, or *in vivo* antitumor activity under deficient experimental conditions). Editors and reviewers of scientific journals, as well as grant decision makers, can play a crucial role in preventing researchers from following this type of approaches. They should consider requesting the *in vitro* and the *in vivo* tests when the goal of the research is to assess preclinical anticancer activity. Negative results obtained under suitable experimental conditions should find a place in research journals. This would encourage researchers to follow solid experimental designs, even though they are unlikely to lead to the desired result. In addition, negative results obtained under suitable experimental conditions can be more valuable than positive results obtained under poor experimental conditions. The publication of a paper demonstrating that a drug candidate has a limited therapeutic potential may prevent hundreds or even thousands of research works from being conducted; this would lead to an important saving of research resources that could be used for better purposes. The publication of a positive result obtained under poor experimental conditions may lead to costly and unnecessary preclinical and clinical research.

Finally, it is important to note that the experimental conditions of these tests are set up to detect drug candidates that at least match the anticancer activity of the existing treatments. This is essential because only these drugs will have a chance of being ultimately used in cancer patients. It is easy to predict that most drug candidates will not pass the tests. An important consequence is that the number of perfect research papers showing a promising compound that kills cancer cells *in vitro* and *in vivo* by targeting a key cancer pathway would dramatically decrease. The aim of researchers and scientific journals involved in cancer drug discovery should not be to publish these perfect papers, but to carry out and disseminate research studies evaluating anticancer activity robustly. This would increase the possibilities of finding better anticancer drugs that we all might need.
